# Characterization of collagen profile in peritoneal metastases of colorectal cancer

**DOI:** 10.1038/s41598-025-05604-x

**Published:** 2025-07-01

**Authors:** P. Villarejo-Campos, S. García Gómez-Heras, S. González-Moreno, S. Qian Zhang, R. Franco-Rodríguez, I. Díaz-Caro, R. Olivera-Salazar, D. García-Olmo, M. García-Arranz

**Affiliations:** 1 Department of Surgery, Fundación Jiménez Díaz University Hospital, Avda. Reyes Católicos, 2, 28040 Madrid, Spain; 2 Department of Surgery, Universidad Autónoma de Madrid, C/Arzobispo Morcillo S/N, 28034 Madrid, Spain; 3 Department of Basic Health Science, Faculty of Health Sciences, Rey Juan Carlos University, 28922 Alcorcón, Madrid, Spain; 4 Department of Surgical Oncology, MD Anderson Cancer Center Madrid, Madrid, Spain; 5 Department of Nursing, Gregorio Marañón University Hospital, Madrid, Spain; 6 New Therapies Laboratory, Health Research Institute-Fundación Jiménez Díaz University Hospital (IIS-FJD), Avda. Reyes Católicos, 2, 28040 Madrid, Spain

**Keywords:** Cancer, Molecular biology

## Abstract

The tumor microenvironment (TME) plays a critical role in cancer progression and response to treatment. In colorectal cancer (CRC), a collagen-rich extracellular matrix (ECM) is a well-established feature of primary tumors and liver metastases. However, the composition of the ECM in peritoneal metastases remains poorly characterized. In this descriptive study, we analyzed the histological distribution of four major collagen types (I, II, III, and IV) in peritoneal metastases from 39 CRC patients using immunohistochemical techniques. Type III fibrillar collagen was predominant, showing moderate to high expression in 80 percent of cases, followed by type IV collagen in 56 percent. Type I collagen demonstrated low intensity in 64 percent of cases. Notably, type II collagen, typically restricted to cartilaginous tissues, was also detected within the tumor stroma, an unexpected finding given its usual absence in this histological contex. These results highlight the unique collagen-rich stroma in CRC peritoneal metastases, dominated by types III and IV collagen, and identify type II collagen as a novel component. This study provides new insight into the stromal architecture of CRC peritoneal metastases and may serve as a foundation for future research on collagen-targeted therapeutic strategies.

## Introduction

Surgical cytoreduction remains the cornerstone treatment for peritoneal metastases in colorectal cancer (CRC). When combined with hyperthermic intraperitoneal chemotherapy (HIPEC), this approach has extended median overall survival to 51 months^1^. However, recurrence rates remain high, with up to 76 percent of patients relapsing within five years^2^, and recent clinical trials have questioned the long-term effectiveness of HIPEC^3^. These findings underscore the need to better identify patients most likely to benefit from cytoreduction and HIPEC^4^, as well as to explore novel therapeutic approaches.

Our group has explored the potential role of collagenase as a novel therapeutic strategy for peritoneal carcinomatosis. We hypothesized that preconditioning the peritoneum with intraperitoneal collagenase could degrade the collagen-rich stroma of peritoneal implants, thereby enhancing chemotherapeutic penetration during HIPEC. In murine models of CRC peritoneal carcinomatosis, this strategy demonstrated promising results^5^, supporting the hypothesis that stromal modulation may improve drug delivery. The collagenase formulation used combined type I (MMP-1) and type II (MMP-8) enzymes, which primarily degrade type I and III collagens^6^. Despite these encouraging findings, a detailed understanding of the extracellular matrix (ECM) composition in CRC peritoneal metastases has been lacking, representing a critical knowledge gap in the rational design of stromal-targeted therapies.

Collagen, the most abundant protein in the human body, plays both structural and signaling roles within tissues. Over 28 collagen types have been identified and are broadly classified as fibrillar or non-fibrillar^7^. Fibrillar collagens (including types I, II, III) assemble into triple-helical fibers, while type IV forms the mesh-like structure of basement membranes. Beyond their structural function, collagens influence a range of biological processes, including cell proliferation, immune modulation, and oxidative stress^8^. In cancer, collagen-rich microenvironments can promote tumor dormancy, immune evasion, and metastatic progression^9–12^.

The ECM is typically organized into two main compartments, the interstitial matrix and the basement membrane, each defined by distinct structural components and collagen profiles. Fibrillar collagens, particularly type I, are predominant in the interstitial matrix, where they provide tensile strength and structural support. In contrast, type IV collagen is a consistent and essential component of the basement membrane across all tissues, playing a critical role in maintaining structural integrity and regulating selective permeability. The specific composition of fibrillar collagens within the interstitial matrix, however, varies depending on the tissue type^13^. For instance, type II collagen predominates in cartilage and the cornea, while types I and III collagens are more abundant in the interstitial matrix of the intestinal mucosa, especially in the colon and rectum^14^. Other fibrillar collagens also contribute to the structural framework of the interstitial matrix^13^. Fibrillar collagens are primarily synthesized by fibroblasts through a combination of intracellular and extracellular mechanisms. In comparison, type IV collagen is synthesized by epithelial and endothelial cells and is essential for establishing the structural and functional integrity of the basement membrane^15^. In the peritoneum, type I collagen forms thick stromal bundles, while type IV collagen is localized to the basement membrane beneath the mesothelium and around the vasculature^16^.

During tumor progression, the ECM undergoes substantial remodeling, leading to the formation of a dense, collagen-rich stroma that promotes tumor invasion and metastasis. In primary CRC, the accumulation of type IV collagen in basement membranes is associated with neoangiogenesis, while the interstitial matrix exhibits increased density of fibrillar collagens. The predominant collagen subtype within the tumor stroma may vary depending on the cancer type^17,18^. In CRC specifically, type I and type III collagens represent the most abundant fibrillar components of the ECM^14^.

The collagen composition of CRC liver metastases has been previously characterized, with type IV collagen identified as the predominant stromal component (Lindgren et al. 2022). In contrast, the collagen composition of peritoneal metastases remains poorly defined.

This study aims to characterize the collagen profile (types I, II, III, and IV) in the ECM of CRC peritoneal metastases using immunohistochemistry. To our knowledge, this is the first descriptive histological analysis of its kind, providing foundational insight into the tumor microenvironment of peritoneal metastases.

## Results

### Sample description

This study analyzed 39 peritoneal metastasis samples (Table 1), derived from colorectal adenocarcinomas (95 percent) and appendiceal adenocarcinomas (5 percent). Among the colorectal cases, the primary tumor was located in the right colon (46 percent), left colon (31 percent), and rectum (18 percent). Most peritoneal metastases (82.1 percent) were metachronous, developing after resection of the primary tumor, while 17.9 percent were synchronous, diagnosed concurrently with the primary tumor. All synchronous metastases originated from right colon (83.3 percent) or appendiceal (16.7 percent) primaries. In contrast, metachronous metastases originated from the appendix (3.1 percent), right colon (40.6 percent), left colon (37.5 percent), and rectum (18.8 percent). A majority of tumors (65.8 percent) were histologically classified as poorly differentiated.

**Table 1:** Clinicopathological characteristics of 39 samples in patients with peritoneal metastases of CRC.

		Time course metastases			
		Synchronous n = 7		Methacronous n = 32	
		Prevalence (%)	Lower–upper limits CI 95%	Prevalence (%)	Lower–Upper limits CI 95%
		17.9	(8.9–32.6)	82.1	(67.3–91.0)
Primary Tumor location	Right colon (includes cecum/ascending colon/hepatic flexure)	83.3	(43.6–96.6)	40.6	(25.5–57.7)
	Left colon (includes descending colon/sigmoid or rectosigmoid junction)			37.5	(22.9–54.7)
	Appendix	16.7	(3.0–56.3)	3.1	(0.5–15.7)
	Rectum			18.8	(8.8–35.3)
Histological Grading	Low grade CRC	42.9	(13.9–76.5)	71.9	(54.9–85.1)
	High grade CRC	57.1	(23.5–86.1)	28.1	(14.9–45.1)
Type of collagen					
Collagen I Intensity	Low	57.1	(23.5–86.1)	65.6	(48.4–80.2)
	Moderate	42.9	(13.9–76.5)	31.3	(17.3–48.4)
	Total	100.0		100.0	
Collagen II Intenity	Low	14.3	(3.9–82.3)	65.6	(48.4–80.2)
	Moderate	28.6	(17.7–96.1)	21.9	(10.4–38.2)
	Not evaluable	57.1	(23.5 – 86.1)	12.5	(4.4 – 27.0)
	Total	100.0		100.0	
Collagen III Intensity	Low	28.6	(6.5–64.8)	15.6	(6.2–30.9)
	Moderate	14.3	(1.6–50.1)	46.9	(30.5–63.8)
	Strong	57.1	(23.5–86.1)	34.4	(19.8–51.6)
	Not evaluable			3.1	(0.3–13.7)
	Total	100.0		100.0	
Collagen IV Intensity	Low	28.6	(6.5–64.8)	40.6	(25.0–57.8)
	Moderate	28.6	(6.5–64.8)	34.4	(19.8–51.6)
	Strong	42.9	(13.9–76.5)	18.8	(8.2–34.6)
	Total	100.0		100.0	

### Collagen expression analysis

**Fig. 1:**
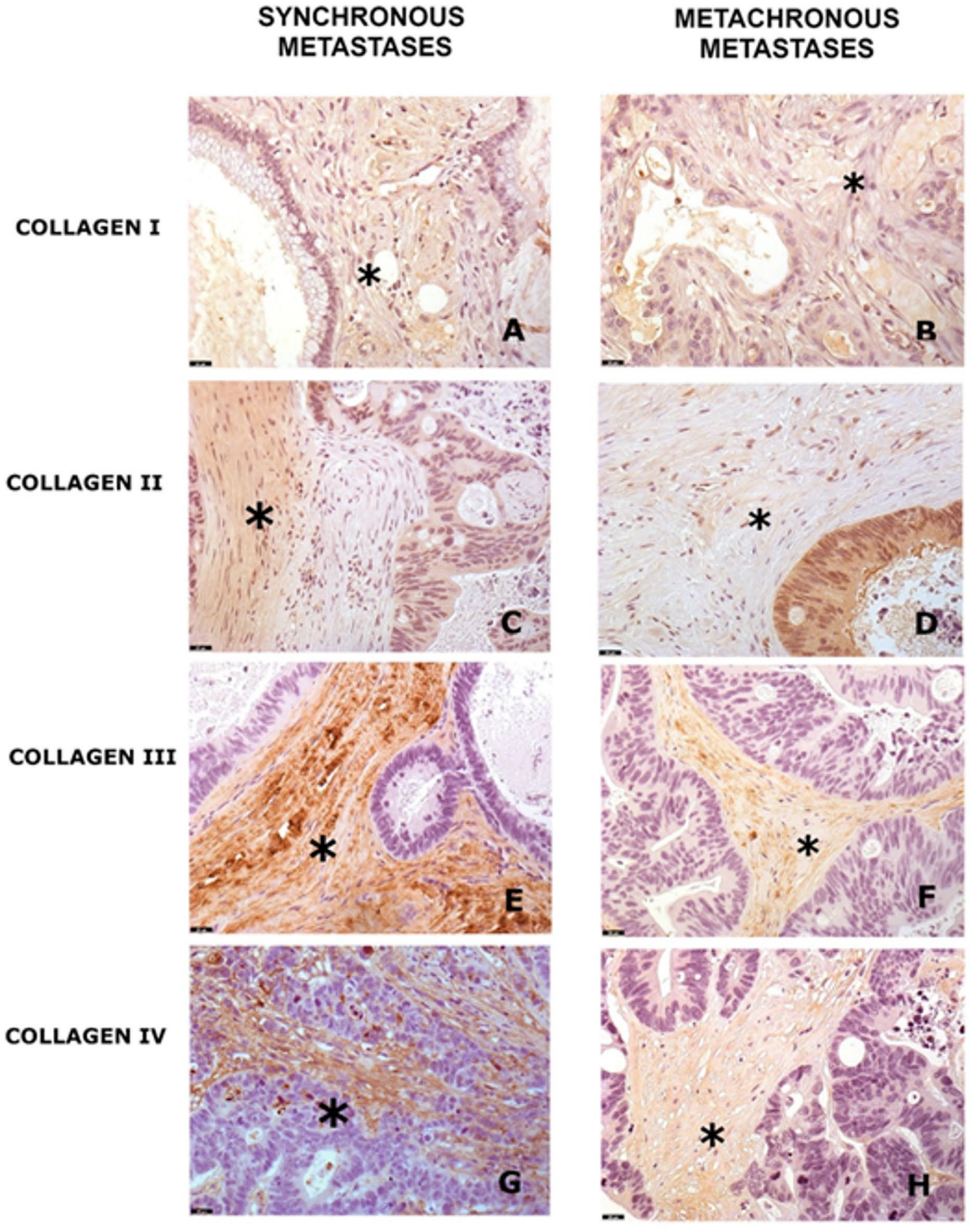
Immunohistochemistry (DAB) analysis results of peritoneal metastases, including synchronous and metachronous cases. Representative images (200× magnification) illustrate collagen expression in the tumor stroma (*). A table at the bottom of the figure summarizes the staining intensities observed across the studied groups.

Immunohistochemical staining revealed marked alterations in collagen fiber organization and distribution within the tumor stroma (Fig. 1). The overall expression profiles of collagens I, II, III, and IV are summarized in Table 1.

Type III collagen was the most prominently expressed, with moderate to high intensity observed in 79.5 percent of the samples. Type IV collagen demonstrated moderate to strong expression in 56.4 percent of cases. Type I collagen was primarily low in intensity, present in expres.1 percent of samples. Interestingly, type II collagen, typically associated with cartilage, was detected at moderate intensity in 23.1percent of peritoneal metastases, representing an unexpected finding in this histological context.

The descriptive analysis of collagen expression according to tumor differentiation grade (low vs. high) did not reveal any clearly distinct patterns. In both groups, type III collagen showed moderate to strong staining in the majority of cases, while collagens I and II were predominantly low in intensity. Type IV collagen exhibited variable expression across both grades, without a consistent trend (Table 2).

**Table 2:** Distribution of collagen expression according to histological grade (high-grade or low-grade) of peritoneal metastatic tissue in CRC patients.

		Low grade CRC n = 26			High grade CRC n = 13			Total n = 39		
		Result n % de N column	95.0% CL Lower Limits % de N column	95.0% CL Upper Limits % de N column	Result n % de N column	95.0% CL Lower Limits % de N column	95.0% CL Upper Limits % de N column	Result n % de N column	95.0% CL Lower Limits % de N column	95.0% CL Upper Limits % de N column
Collagen I Intensity	None	0 (0.0)			0 (0.0)			0 (0.0)		
	Low	18 (69.2)	50.2	84.2	7 (53.8)	28.3	77.9	25 (64.1)	48.5	77.7
	Moderate	7 (26.9)	12.9	45.7	6 (46.2)	22.1	71.7	13 (33.3)	20.1	48.9
	Strong	0 (0.0)			0 (0.0)			0 (0.0)		
	Not evaluable	1 (3.8)	0.4	16.6	0 (0.0)			1 (2.6)	0.3	11.4
	Total	26 (100.0)			13 (100.0)			39 (100.0)		
Collagen II Intenity	None	0 (0.0)			0 (0.0)			0 (0.0)		
	Low	16 (61.5)	42.4	78.2	6 (46.2)	22.1	71.7	22 (56.4)	40.9	71.1
	Moderate	4 (15.4)	5.4	32.5	5 (38.5)	16.5	65.0	9 (23.1)	12.1	37.9
	Strong	0 (0.0)			0 (0.0)			0 (0.0)		
	Not evaluable	6 (23.1)	10.3	41.5	2 (15.4)	3.3	40.9	8 (20.5)	10.2	35.0
	Total	26 (100.0)			13 (100.0)			39 (100.0)		
Collagen III Intensity	None	0 (0.0)			0 (0.0)			0 (0.0)		
	Low	7 (26.9)	12.9	45.7	0 (0.0)			7 (17.9)	8.4	32.0
	Moderate	10 (38.5)	21.8	57.6	6 (46.2)	22.1	71.7	16 (41.0)	26.7	56.6
	Strong	9 (34.6)	18.7	53.7	6 (46.2)	22.1	71.7	15 (38.5)	24.5	54.1
	Not evaluable	0 (0.0)			1 (7.7)	0.8	30.7	1 (2.6)	0.3	11.4
	Total	26 (100.0)			13 (100.0)			39 (100.0)		
Collagen IV Intensity	None	0 (0.0)			0 (0.0)			0 (0.0)		
	Low	11 (42.3)	25.0	61.3	4 (30.8)	11.4	57.7	15 (38.5)	24.5	54.1
	Moderate	9 (34.6)	18.7	53.7	4 (30.8)	11.4	57.7	13 (33.3)	20.1	48.9
	Strong	5 (19.2)	7.7	37.1	4 (30.8)	11.4	57.7	9 (23.1)	12.1	37.9
	Not evaluable	1 (3.8)	0.4	16.6	1 (7.7)	0.8	30.7	2 (5.1)	1.1	15.4
	Total	26 (100.0)			13 (100.0)			39 (100.0)		

## Discussion

Solid tumors are characterized by a substantial proportion of stromal tissue, which may comprise up to 70 percent of the total tumor mass^20^. This stromal component, present in both primary and metastatic tumors, is composed of cells and protein structures such as collagen, which play critical roles in cancer progression, tumor cell invasiveness, survival, and resistance to treatment^21,22^.

The role of collagen in the TME extends beyond structural support; it actively participates in direct communication with cancer cells within the ECM. This interaction triggers multiple signaling pathways and complex molecular mechanisms that promote tumor progression and metastasis^23^.

In colorectal adenocarcinoma, the composition and organization of collagen in the TME have been extensively studied and are closely linked to invasive behavior^24,25^. In primary CRC tumors, types I and III collagens are the most abundant within the stroma. Interestingly, type IV collagen is elevated in tumors without distant spread but tends to decline in cases with established distant metastases^24^.

The collagen composition of metastatic tissues, such as liver metastases of colorectal origin, has been described, with type IV collagen identified as the predominant stromal component^26^. However, these patterns remain unexplored in peritoneal metastases. This study addresses that gap by demonstrating that, in peritoneal metastases of colorectal origin, type III and type IV collagens are the most abundant within the tumor stroma. Additionally, we identified type II collagen, an unexpected finding in this context, as it is typically synthesized by chondrocytes^27^.

The presence of type II collagen in the tumor stroma of peritoneal metastases, is noteworthy. While type II collagen is primarily associated with cartilage, where it helps maintain tissue integrity, recent studies suggest that the NH2-propeptide of type IIB collagen can induce tumor cell apoptosis and inhibit invasion through integrin-mediated interactions^28^. Its detection in peritoneal metastases suggests a potential, albeit speculative, modulatory role in tumor progression.

Premetastatic tumor niches are characterized by an ECM rich in type III collagen, which plays an active role in inducing and maintaining tumor cell dormancy^10^. Type III collagen promotes the undulation and disorganization of collagen fibers in the tumor stroma, a feature associated with metastatic tumor cell dormancy. During dormancy, tumor cells remain inactive, but this state is reversible. Overexpression of type III collagen in the TME is regulated by the activation of Discoidin Domain Receptor 1 (DDR1), a receptor tyrosine kinase, via STAT1 signaling^10,29^. These findings raise the possibility that the predominance of type III collagen in colorectal peritoneal metastases may be linked to tumor dormancy mechanisms, similar to those described in premetastatic niches.

A collagen-rich tumor stroma, particularly abundant in type IV collagen, has also been described in liver metastases of colorectal origin^26^. The elevated expression of type IV collagen in the TME of metastases seems to correlate with the prognosis and aggressiveness of the metastatic process^30^. Our results similarly underscore the importance of ECM composition in defining metastatic behavior.

Taken together, our findings support the need to further characterize collagen profiles in peritoneal metastases, particularly in light of therapeutic strategies aimed at modifying the ECM. The selection of appropriate collagenase types, tailored to the predominant collagen subtypes, may be critical for optimizing such approaches. Preclinical studies have already demonstrated improved HIPEC efficacy following sequential intraperitoneal collagenase administration^5^ support continued investigation of this approach as a potential therapeutic tool in the future.

## Methods

### Study design

This study investigates the histological profile of key collagen types (I, II, III, and IV) in the stroma of peritoneal metastases of colorectal origin. This was a descriptive, observational, and retrospective study involving 39 samples of peritoneal metastases derived from colorectal and appendiceal adenocarcinomas. The samples were sourced from the tissue biobank of the Anatomic Pathology Service at Fundación Jiménez Díaz Hospital and MD Anderson Cancer Center in Madrid, Spain. These samples were collected from patients who underwent surgery between February 15, 2001, and March 19, 2021. Ethical approval for the study was granted in January 2021 by the Ethics Committee of Fundación Jiménez Díaz (code: PIC003-21_FJD). All samples of peritoneal metastases were donated and stored in the biobanks for research purposes. Patients provided specific written informed consent for the use of their samples in a coded form for research studies.

### Histopathological analysis

Samples of peritoneal metastasis (5 mm^3^) were fixed in 10 percent of formaldehyde at room temperature, embedded in paraffin, and sectioned into 5-micron-thick slices using a Micron HM360 microtome.

The sections were initially stained with hematoxylin and eosin to assess the tumor characteristics. To analyze the intensity and distribution of collagen expression, immunohistochemical staining was performed as follows: the sections were deparaffinized, rehydrated, and the endogenous peroxidase activity was blocked using 0.3 percent of hydrogen peroxide (H₂O₂) in methanol. After washing with phosphate-buffered saline (PBS), the slides were incubated with primary antibodies in a moist chamber at room temperature. The sections were then incubated with biotinylated anti-rabbit IgG and LBA (DAKO) for 25 min, followed by PBS washing and immersion in avidin-peroxidase for another 25 min. The immunostaining reaction product was developed using diaminobenzidine (DAB), and counterstaining was performed with hematoxylin. The specificity of the staining was confirmed by incubating some sections with non-immune serum instead of the primary antibody.

The primary antibodies used for immunohistochemistry were: anti-collagen I (Abcam ab34710), anti-collagen II (Abcam ab34712), anti-collagen III (Abcam ab6310), and anti-collagen IV (Abcam ab6586).

The histological slides were examined under a Zeiss Axiophot 2 microscope, and images were captured using an AxiocamHRc camera. The negative controls for the anti-collagen antibodies used in this study are available in the supplementary material (Supplementary figure S1). All evaluations were conducted by the same researcher, who was blinded to the clinical details of the samples.

### Semiquantitative evaluation and scoring of collagen expression

The immunohistochemically stained tissue sections were evaluated and scored according to the methods previously described by Wei et al.^31^ and Huang et al^32^. The evaluation criteria were as follows:

*Intensity scoring*: 0 (negative), 1 (weak), 2 (moderate), and 3 (strong).

*Extent of staining*: 0 (0 percent), 1 (1 to 25 percent), 2 (26 to50 percent), 3 (51 to 75 percent), and 4 (76 to100 percent), based on the percentage of positive staining relative to the total carcinoma area.

In this study, two sections from each tumor were analyzed. All evaluations were conducted by a single researcher blinded to the clinical details of the samples, ensuring objectivity, consistency, and reproducibility in the assessment.

### Statistical analysis

This study was designed as a descriptive analysis of collagen expression patterns in peritoneal metastases. Statistical analysis was performed using SPSS version 25 (Windows). Qualitative variables were described using absolute frequencies and percentages.

To assess prevalence and estimate confidence limits (lower and upper) the EPITOOLS© epidemiological calculator was used. This tool calculates the estimated proportion and its corresponding 95 percent confidence interval using the Wilson method, as described by Brown, LD et al.^33^.

### Limitations

This study has several limitations that should be acknowledged. First, the sample size was relatively small (n = 39), and the subgroup of synchronous metastases was limited. This precluded the use of robust inferential statistical analyses and restricted the ability to draw definitive conclusions about differences between subgroups. Second, the study was purely descriptive and based exclusively on immunohistochemical techniques, without complementary molecular assays to further explore the biological significance of the observed collagen patterns.

Finally, while the detection of type II collagen is a novel and intriguing finding, its functional implications remain speculative and warrant further investigation. Despite these limitations, this study provides an important preliminary step toward characterizing the extracellular matrix in peritoneal metastases of colorectal origin, and it may inform future research on stromal targeting as a therapeutic strategy.

## Supplementary Information

Supplementary Information.

## Data Availability

All data generated or analysed during this study are included in this published article.
