# Supplementary material for: Characterization of collagen profile in peritoneal metastases of colorectal cancer
Source: Sci Rep. 2025 Jul 1;15:20528. doi: 10.1038/s41598-025-05604-x (PMC12218282; doi:10.1038/s41598-025-05604-x)
Supplement: Supplementary file 1 — Supplementary Information. [file 41598_2025_5604_MOESM1_ESM.docx]

Supplementary Figure 1: Negative controls of anti-collagen antibodies. Immunohistochemistry DAB (200× magnification)


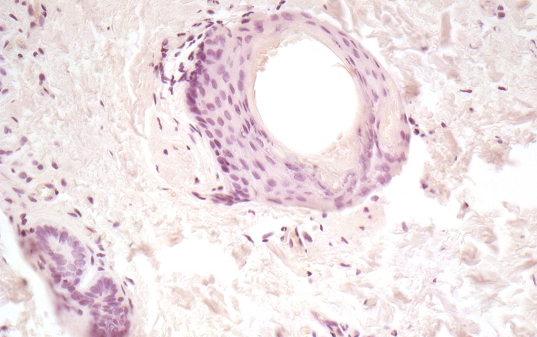

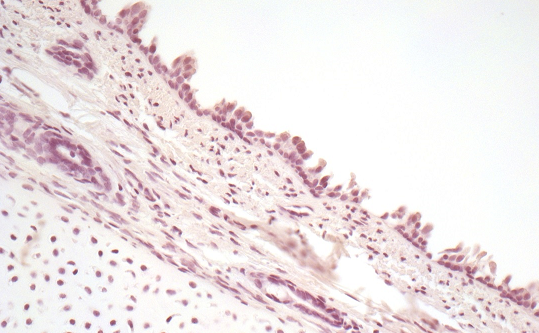


**A**

**B**

A: Negative control anti-collagen I antibody (Abcam ab34710). B: Negative control anti-collagen II antibody (Abcam ab34712).

Human hair follicle. Human bronchus.


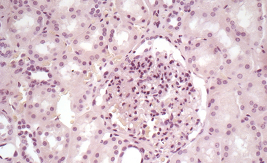

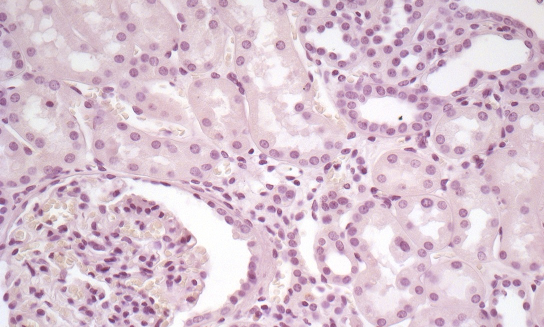


**D**

**C**

C: Negative control anti-collagen III antibody (Abcam ab6310). D: Negative control anti-collagen IV antibody (Abcam ab6586).

Human kidney. Human kidney.
